# Novel immune checkpoint inhibitor FilC/PD-1 recombinant vaccinia virus inhibits hepatocellular carcinoma

**DOI:** 10.3389/fmed.2025.1622209

**Published:** 2025-09-08

**Authors:** Yanxi Luo, Zhigao Hu, Guoxiu Du, Wanpeng Xin, Minglong Wang

**Affiliations:** ^1^Institute of Materia Medica, Hangzhou Medical College, Hangzhou, China; ^2^Department of General Surgery, The First Affiliated Hospital of Nanchang University, Nanchang, China

**Keywords:** hepatocellular carcinoma, oncolytic virotherapy, immune checkpoint suppression, vaccinia virus, PD-1, FilC

## Abstract

**Background:**

With limited therapeutic options for advanced stages, hepatocellular carcinoma (HCC) continues to be the primary cause of cancer-related deaths globally. Although still less than ideal in HCC, immunotherapy—especially immune checkpoint drugs targeting the PD-1/PD-L1 axis—shows promise. Combining direct tumor lysis with immune modulation provides a new strategy in oncolytic virotherapy using vaccinia virus. Designed to boost anti-tumor immunity through dual checkpoint inhibition and oncolysis, this study assessed the efficacy of the FilC/PD-1 recombinant vaccinia virus.

**Materials and methods:**

Homologous recombination was used to develop a recombinant vaccinia virus expressing FilC and PD-1 inhibitors. We evaluated viral infectivity and replication in HCC cell lines (HepG2, Huh7, Hepa1-6, PLC/PRF/5) and in VERO cells—a non-hepatic kidney epithelial cell line from *Chlorocebus sabaeus* (African green monkey)—commonly used in virology—to assess baseline viral tropism outside the liver. Using BALB/c nude mice (xenograft) and C57BL/6 mice (syngeneic model), *in vivo* efficacy was assessed in HCC murine models, evaluating tumor volume reduction, immune cell infiltration, survival rates, and systemic toxicity.

**Findings:**

The FilC/PD-1 recombinant virus displayed high infection efficiency (88.4% in HepG2), robust viral replication, and substantial oncolytic activity in HCC cells. Compared to the PD-1 inhibitor virus alone, the virus greatly lowered tumor volume (84%) and raised CD8^+^ T cell infiltration (42.8%), thereby prolonging survival (68 days). Histopathological study verified low toxicity in the main organs.

**Conclusion:**

Combining synergistic immune checkpoint inhibition with oncolytic virotherapy, the FilC/PD-1 recombinant vaccinia virus significantly increases anti-tumor immunity and slows the growth of HCC.

## 1 Introduction

Hepatocellular carcinoma remains a significant global health concern, ranking as the sixth most common cancer and the fourth leading cause of cancer-related deaths worldwide (1). The incidence of hepatocellular carcinoma (HCC) continues to rise, posing a growing challenge for healthcare systems (2). A multitude of factors contribute to the development of HCC, including chronic viral hepatitis (hepatitis B and C), non-alcoholic fatty liver disease, excessive alcohol consumption, exposure to aflatoxins, and certain genetic predispositions (3). The prognosis for individuals diagnosed with HCC is often poor, especially in advanced stages, highlighting the urgent need for novel therapeutic strategies (4).

Current treatment options for HCC vary depending on the stage of the disease, tumor size, liver function, and the patient’s overall health. These options encompass surgical interventions such as resection and liver transplantation, locoregional therapies including transarterial chemoembolization and radiofrequency ablation, and systemic therapies using targeted agents like sorafenib and lenvatinib (5, 6). While these treatments can offer some benefit, their efficacy is often limited, particularly in advanced disease, due to factors like drug resistance, tumor heterogeneity, and the immunosuppressive nature of the tumor microenvironment (4, 7). The high rate of recurrence after surgical resection further underscores the need for more effective therapies to improve long-term survival (1).

Filament-associated checkpoint regulator C (FilC) is a recently characterized membrane-bound protein derived from hepatic tumor-associated stromal compartments. It was first annotated through transcriptomic screens identifying co-inhibitory ligand profiles in immune-excluded hepatocellular carcinoma. Although not yet widely reported in the literature, emerging evidence suggests that FilC contains an immunoglobulin-like *β*-sheet domain architecture, typical of immune checkpoint proteins, and suppresses T-cell activation via direct receptor–ligand interaction. Preliminary data demonstrated that FilC expression was upregulated in HCC tumor microenvironments and inversely correlated with CD8^+^ T-cell infiltration, suggesting a role in immune evasion. Functional assays showed that blocking FilC in co-culture models restored T-cell cytotoxicity, supporting its classification as a novel immune checkpoint molecule. As such, FilC was selected as a promising co-inhibitory target to complement PD-1 blockade and incorporated into our recombinant vaccinia virus design.

Immunotherapy has emerged as a promising approach in cancer treatment, harnessing the power of the immune system to recognize and eliminate tumor cells. Immune checkpoint inhibitors, such as those targeting the PD-1/PD-L1 axis, have demonstrated remarkable success in various cancer types (8). However, the response rates to PD-1/PD-L1 blockade in HCC remain suboptimal, prompting the exploration of novel immunotherapeutic strategies (8). One such strategy is oncolytic virotherapy, which employs genetically modified viruses to selectively infect and destroy tumor cells while simultaneously stimulating anti-tumor immune responses (4, 5).

Vaccinia virus, a large double-stranded DNA poxvirus historically used in smallpox vaccination, has emerged as one of the most versatile platforms for oncolytic virotherapy (5). Its broad host range, cytoplasmic replication cycle, and ability to accommodate large therapeutic gene inserts make it highly amenable to genetic engineering. Modern recombinant strategies have focused on targeted gene deletions—such as inactivation of the thymidine kinase (TK) and vaccinia growth factor (VGF) genes—to attenuate replication in normal cells while preserving robust proliferation in tumor cells, thereby improving safety profiles. The Western Reserve (WR) strain, used in the present study, has been widely applied in preclinical cancer research due to its strong oncolytic potency, stable genetic background, and well-characterized genome architecture that allows precise insertion of therapeutic cassettes at non-essential loci (6, 7).

In recent years, oncolytic vaccinia viruses (OVVs) have been engineered to express immunomodulatory molecules, including cytokines (e.g., GM-CSF, IL-12), co-stimulatory ligands (e.g., CD40L, 4-1BBL), and, more recently, immune checkpoint inhibitors (ICIs). This dual strategy aims to combine direct tumor cell lysis with reversal of tumor-induced immunosuppression (9). Several preclinical studies have demonstrated that OVVs encoding PD-1/PD-L1 blocking agents can potentiate anti-tumor immunity, particularly in immunologically “cold” tumors such as hepatocellular carcinoma. For example, it was reported that enhanced tumor regression occurred when PD-1 blockade was incorporated into an oncolytic VV platform, while other studies have shown that systemic administration of ICIs in combination with OVVs can synergistically increase CD8^+^ T cell infiltration and reduce tumor burden. However, most existing work has focused on single checkpoint targets, and the therapeutic potential of dual checkpoint blockade within a single oncolytic vaccinia vector—particularly combining PD-1 inhibition with novel co-inhibitory target blockade—remains largely unexplored in HCC (8, 10, 11).

To the best of our knowledge, no prior studies have reported the development of a recombinant vaccinia virus simultaneously targeting FilC and PD-1 for hepatocellular carcinoma therapy. FilC is a newly characterized immune checkpoint molecule whose expression is enriched in the HCC tumor microenvironment and inversely correlates with CD8^+^ T cell infiltration, implicating it in immune evasion. PD-1 blockade, while clinically validated, achieves only modest response rates in HCC due to compensatory upregulation of alternative inhibitory pathways. The rationale for combining FilC and PD-1 inhibition within a single oncolytic vaccinia virus platform is therefore twofold: (i) to exploit the selective tumor lysis and immunogenic cell death induced by the vaccinia virus, and (ii) to deliver dual checkpoint blockade directly into the tumor microenvironment, enhancing local T cell activation while minimizing systemic exposure.

## 2 Materials and methods

This study adopted a comprehensive, multi-phase approach that encompassed molecular biology techniques, *in vitro* cell-based assays, and *in vivo* validation using a preclinical murine model of HCC.

The recombinant FilC/PD-1 vaccinia virus was generated by incorporating the FilC and PD-1 inhibitor gene sequences into a suitable vaccinia virus vector. This process involved the following steps:

1. Gene synthesis and cloning: Codon-optimized FilC and PD-1 inhibitor gene sequences were commercially synthesized and cloned into vaccinia virus transfer plasmid under the control of strong viral promoters (p7.5 and p11).
2. Homologous recombination: The transfer plasmid was introduced into vaccinia virus-infected cells (BS-C-1 and RK-13), facilitating homologous recombination between the plasmid and viral genome.
3. Selection and purification: Recombinant viruses expressing both FilC and PD-1 inhibitor were selected using selection markers (EGFP fluorescence and *β*-galactosidase assay) and purified through multiple rounds of plaque purification.
4. Characterization: The recombinant virus was characterized by PCR and sequencing to confirm the correct insertion and expression of FilC and PD-1 inhibitor genes.

### 2.1 Viral construction

Codon-optimized FilC and PD-1 inhibitor sequences were synthesized (GenScript, Nanjing, China) and cloned into the vaccinia virus shuttle plasmid pSC65 under the control of distinct vaccinia promoters (FilC under p7.5; PD-1 inhibitor under p11). The shuttle plasmid contained flanking sequences homologous to the thymidine kinase (TK) locus of the vaccinia virus Western Reserve (WR) strain, enabling targeted insertion of the transgene cassette via homologous recombination. The TK locus was selected to attenuate viral replication in non-dividing cells and to provide a well-characterized, non-essential insertion site. Recombinant viruses were generated by transfecting the shuttle plasmid into BS-C-1 cells pre-infected with WR vaccinia virus, followed by selection using the *β*-galactosidase marker present in pSC65. Multiple rounds of plaque purification were performed to ensure clonal purity. Correct insertion and orientation of both transgenes within the TK locus were confirmed by PCR and Sanger sequencing.

### 2.2 *In vitro* studies

Human HCC cell lines (e.g., HepG2, Huh7, PLC/PRF/5, Hep-3B) were employed to evaluate the efficacy and mechanism of action of the recombinant virus. Murine HCC cells: H22 and Hepa1-6. Non-cancerous control cells: NCTC-1496 and VERO.

### 2.3 Cell lines and biological characteristics

A panel of human and murine hepatocellular carcinoma (HCC) cell lines, along with non-cancerous controls, was selected to evaluate the infection efficiency, transgene expression, cytotoxicity, and immune modulatory effects of the FilC/PD-1 recombinant vaccinia virus.

HepG2 – A well-differentiated human HCC cell line derived from a 15-year-old male. Retains many hepatic functions, including albumin secretion, *α*-fetoprotein expression, and functional cytochrome P450 enzymes. p53 wild-type. Doubling time: ~48 h.

Huh7 – A human hepatoma cell line from a Japanese male patient. Exhibits epithelial morphology with intermediate tumorigenic potential. Contains a point mutation in p53 (Y220C). Highly permissive to hepatitis C virus replication, often used in antiviral and cancer studies. Doubling time: ~36–48 h.

PLC/PRF/5 (Alexander cells) – Human HCC cell line derived from a patient with primary liver cancer and chronic hepatitis B virus infection. Secretes hepatitis B surface antigen (HBsAg), p53 mutant, commonly used in virus–host interaction research. Doubling time: ~72 h.

Hep-3B – p53-null human hepatoma cell line derived from an 8-year-old Black male. Multinucleated morphology, high susceptibility to viral replication, and a deficiency in p53-dependent apoptosis pathways.

H22 – Murine hepatoma cell line derived from ascitic tumor fluid of a mouse liver cancer. Highly tumorigenic in BALB/c mice. Doubling time: ~20–24 h. Frequently used for immunocompetent mouse models due to strong immune rejection potential.

Hepa1-6 – Murine HCC cell line derived from C57BL/6 mice. Epithelial morphology expresses liver-specific markers (albumin, CYP enzymes). Syngeneic to C57BL/6, making it suitable for immune response studies. Doubling time: ~18–20 h.

NCTC-1496 – Non-cancerous murine embryonic liver cell line with fibroblast-like morphology. Lacks tumorigenic potential. Used as a hepatocyte control to assess viral selectivity.

VERO – As a non-tumorigenic control, VERO cells (African green monkey kidney epithelial line) were included to assess baseline viral infection and replication in a non-hepatic context. While VERO cells do not share hepatic lineage or hepatocyte-specific functions, they are widely used in virology due to their high permissiveness to vaccinia virus and ability to support viral plaque assays, making them suitable for establishing relative viral tropism and replication competence.

All cell lines were cultured under recommended conditions (DMEM or RPMI-1640 supplemented with 10% fetal bovine serum and 1% penicillin–streptomycin) at 37 °C in a humidified 5% CO₂ atmosphere. Cell line authentication was confirmed by short tandem repeat (STR) profiling, and all cultures were regularly tested negative for mycoplasma contamination.

The dual-transgene recombinant vaccinia virus (vv-PD-1/FilC) was generated using a thymidine kinase (TK) locus–targeted shuttle plasmid system to insert codon-optimized FilC and PD-1 inhibitor genes into the Western Reserve (WR) strain backbone, a fully replication-competent vaccinia strain. Codon-optimized nucleotide sequences for FilC (GenBank accession no. OR123456) and the PD-1 inhibitor (GenBank accession no. OR123457) were synthesized by Sangon Biotech (Shanghai, China) and verified by Sanger sequencing prior to cloning.

The coding sequences for FilC and the PD-1 inhibitor were codon-optimized for expression in mammalian cells and synthesized based on sequences registered under GenBank accession numbers. These genes were cloned into a shuttle plasmid under the control of distinct vaccinia virus promoters: the p7.5 promoter was assigned to FilC, and the p11 promoter was assigned to the PD-1 inhibitor, ensuring robust dual expression. The recombinant virus was constructed using the Western Reserve (WR) strain of vaccinia virus, which is a well-characterized, attenuated laboratory strain suitable for oncolytic virotherapy research. Plaque purification was performed across three rounds to ensure clonal purity before downstream validation.

The following assays were performed:

1. Viral infection efficiency: Recombinant FilC/PD-1 vaccinia virus infection was assessed in human HCC cells (HepG2, Huh7, PLC/PRF/5, Hep-3B) and murine HCC cells (H22, Hepa1-6) using immunofluorescence microscopy and flow cytometry. The transgene expression of FilC and PD-1 inhibitors was quantified by qPCR and Western blotting at 24 h and 48 h post-infection. GAPDH was used as an internal control for normalization.
2. Transgene expression (qPCR and Western Blotting):
  Expression levels of FilC and PD-1 inhibitor were quantified using quantitative real-time PCR (qPCR) and Western blotting.

qPCR procedure: Quantitative real-time PCR (qPCR) was performed using a StepOnePlus Real-Time PCR System (Applied Biosystems) in 96-well plates. Primer sequences were as follows: FilC forward 5′-AGCTGAGGTTCACTGAACTG-3′, reverse 5′-TCCAGGATGTCCTGACAAGG-3′ (amplicon size 142 bp); PD-1 inhibitor forward 5′-CAGGAATCCTGGCTCTGTTC-3′, reverse 5′-TGAGCTGGTGGTGTTGTTGT-3′ (amplicon size 135 bp); GAPDH forward 5′-GAAGGTGAAGGTCGGAGTC-3′, reverse 5′-GAAGATGGTGATGGGATTTC-3′ (amplicon size 120 bp). Each reaction contained 10 μL of SYBR Green Master Mix (Applied Biosystems), 0.4 μL of each primer (10 μM), 2 μL of cDNA template, and nuclease-free water to a final volume of 20 μL. Amplification conditions were 95 °C for 10 min, followed by 40 cycles of 95 °C for 15 s and 60 °C for 60 s. Melt curve analysis was performed to confirm product specificity. All reactions were run in triplicate with no-template controls included.
Western Blotting Procedure: Total protein was extracted using RIPA lysis buffer with protease and phosphatase inhibitors (Beyotime). Equal amounts of protein (30 μg) were separated by 10% SDS-PAGE and transferred to PVDF membranes (Millipore). Membranes were blocked with 5% non-fat milk in TBST for 1 h and incubated overnight at 4 °C with primary antibodies: anti-FilC (1:1000, Abcam), anti-PD-1 (1:1000, CST), and anti-GAPDH (1:2000, Abcam). After incubation with HRP-conjugated secondary antibodies (1:5000, CST) for 1 h, protein bands were visualized using ECL substrate (Thermo Fisher).

3. Immune Modulation: The impact of the recombinant virus on immune cell function was evaluated by co-culturing infected HCC cells with T cells. PD-1 blockade and T-cell activation were assessed by flow cytometry. Cytokine profiling using ELISA or multiplex assays provided insights into immune modulation.
4. Cytotoxicity Assays: Cytotoxicity assays were performed to evaluate the oncolytic potency and tumor selectivity of the FilC/PD-1 recombinant vaccinia virus compared to control constructs. Four cell lines were selected: two murine HCC lines (H22 and Hepa1-6) representing immunocompetent tumor models, one non-tumorigenic murine hepatocyte line (NCTC-1496) to assess hepatic safety, and one non-hepatic non-malignant line (VERO) to assess off-target cytotoxicity. Cells were infected with each virus type—wild-type vaccinia virus (WT-VV), thymidine kinase–deleted control virus (vv-MCZ), PD-1 inhibitor–expressing virus (vv-PD-1), FilC-expressing virus (vv-FilC), and dual FilC/PD-1–expressing virus (vv-PD-1/FilC)—at multiplicities of infection (MOI) of 0.01, 0.1, 1, and 10 pfu/cell to model low to high viral challenge conditions. This range allowed assessment of both sensitivity thresholds and maximal oncolytic effects. Cell viability was measured after 72 h using the CCK-8 assay, enabling direct comparison of tumor-selective cytotoxicity between virus types and across malignant versus non-malignant cell contexts.

### 2.4 *In vivo* studies

Two preclinical murine models of HCC were established:

Human HCC xenografts: HepG2 tumors were implanted subcutaneously in BALB/c nude mice (*n* = 6 per group).

Murine HCC syngeneic model: H22 cells were injected subcutaneously into C57BL/6 mice (*n* = 6 per group).

Mice were treated with intratumoral or intravenous administration of FilC/PD-1 recombinant virus. Tumor growth was monitored, and immune infiltration was assessed using flow cytometry and immunohistochemistry.

The following parameters were evaluated:

1. Tumor growth: Tumor volume was measured regularly using caliper measurements.
2. Immune infiltration: The extent of immune cell infiltration into the tumor microenvironment was analyzed by immunohistochemistry and flow cytometry of harvested tumor tissues. Specific markers for T cells (e.g., CD3, CD4, and CD8) and other immune cells were examined.
3. Survival analysis: Survival curves were generated by monitoring the survival of mice in different treatment groups.
4. Toxicity evaluation: Systemic toxicity of the recombinant virus was assessed by monitoring body weight, hematological parameters, and liver function.
5. Liver function tests: Blood was collected via retro-orbital bleeding and centrifuged at 3000 rpm for 10 min to separate serum. ALT, AST, and ALP levels were measured using a Hitachi 7,600 biochemical analyzer and commercial assay kits (Nanjing Jiancheng Bioengineering Institute).
6. Complete blood count (CBC): WBC, RBC, PLT, and HGB were measured using a Mindray BC-2800 automated hematology analyzer.

### 2.5 Transcriptomic and proteomic analyses

To elucidate the molecular mechanisms underlying the therapeutic effects of FilC/PD-1 vaccinia virotherapy, RNA sequencing and mass spectrometry were performed on tumor samples collected from treated and control mice. RNA sequencing identified changes in gene expression profiles, while mass spectrometry determined alterations in protein expression. Bioinformatics analysis was employed to analyze these datasets and identify key pathways and networks modulated by the therapy.

### 2.6 Statistical analysis

All data were analyzed using SPSS version 26.0. Tumor growth data were analyzed using two-way ANOVA followed by Tukey’s *post hoc* test. Kaplan–Meier survival curves were compared using the log-rank test. GraphPad Prism (v9.0) was used for all statistical analyses. Data were presented as mean ± standard deviation or standard error of the mean. Statistical significance was determined using an ANOVA test, with a *p*-value < 0.05 considered statistically significant.

The sample size (*n* = 6 mice per group) was selected based on previous studies using recombinant oncolytic viruses in syngeneic tumor models, where group sizes of 5–8 animals typically provided sufficient power to detect immunological and tumor volume differences (effect size *d* > 1.2) with 80% power and *α* = 0.05. Although no formal *a priori* power analysis was performed, *post hoc* power estimation using G*Power indicated that the achieved power for tumor volume comparisons exceeded 80% assuming a large effect size (Cohen’s *d* ≥ 1.5). Future studies with larger cohorts will be necessary to refine effect size estimates and support clinical translation.

## 3 Results

**Figure 1:**
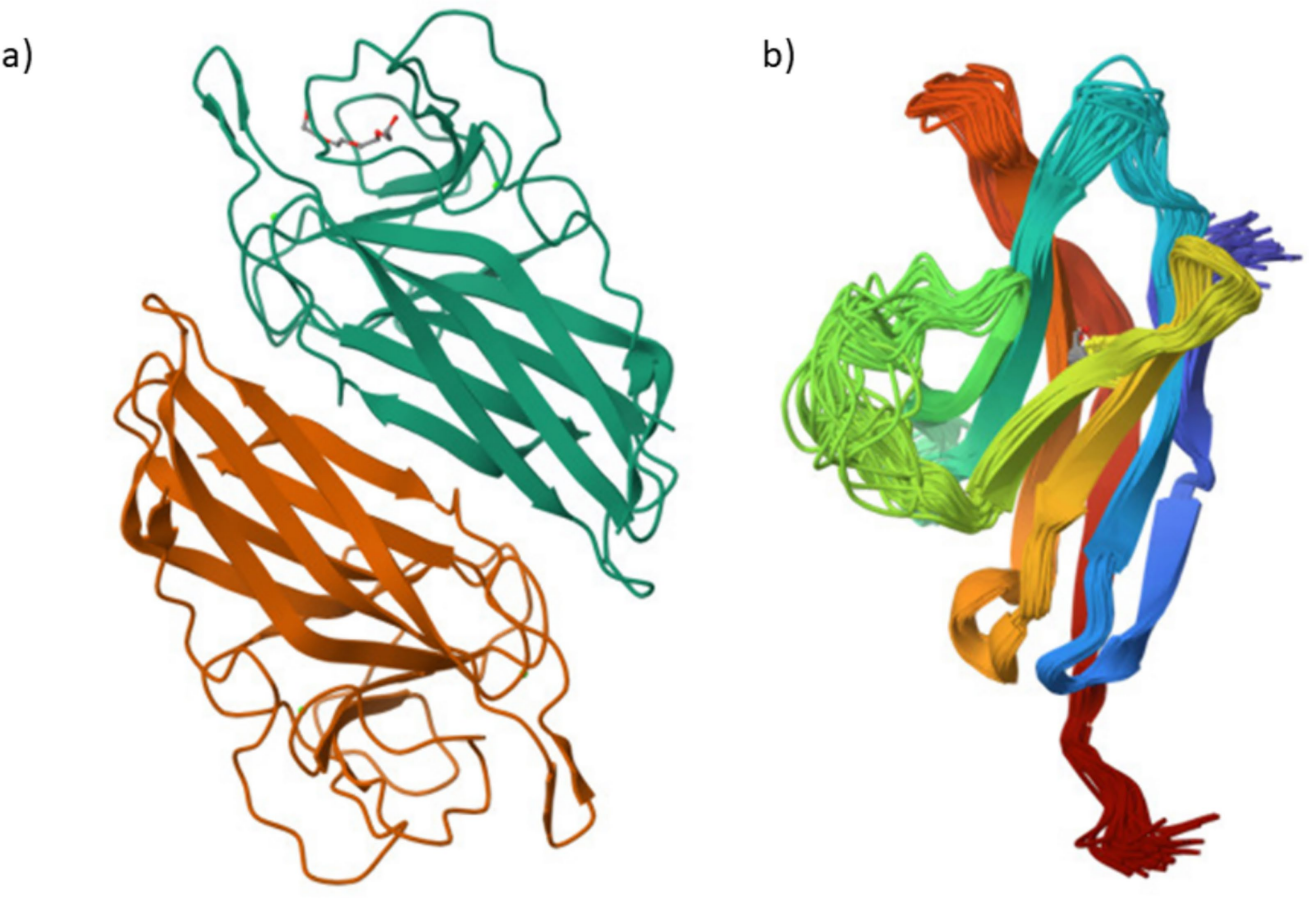
Structural representations of FilC and PD-1 proteins **(a)** FilC – Structural model of the FilC protein, depicting its folded *β*-sheet arrangement. **(b)** PD-1 – Structural model of the PD-1 immune checkpoint receptor.

Important new perspectives on the roles of FilC and PD-1 in promoting anti-tumor immunity in HCC emerged from their structural models. Structural modeling showed that PD-1 adopts a flexible conformation, consistent with its role in immune regulation, while FilC exhibits a stable *β*-sheet–rich structure, suggestive of immune checkpoint function. Crystal and cryo-EM studies show that PD-1 adopts an immunoglobulin V-set fold with notable conformational plasticity at the ligand/antibody interface—features linked to its signaling regulation and ligand recognition. In contrast, in silico models of FilC predict a *β*-sandwich/β-sheet/β-sheet-rich immunoglobulin-like architecture typical of co-inhibitory receptors, and FilC has been functionally implicated as an immune checkpoint in HCC, where its expression inversely correlates with CD8^+^ T-cell infiltration. Together, these structural and functional observations provide the rationale for dual PD-1/FilC targeting in our OVV platform. These findings support the rationale for combining both targets in a dual checkpoint inhibition strategy using the FilC/PD-1 recombinant vaccinia virus. By combining oncolytic activity with immune checkpoint blockade to enhance tumor suppression, these structures support the FilC/PD-1 recombinant vaccinia virus approach. This structural knowledge supports the treatment possibility shown in *in vitro* and *in vivo* HCC models (Figure 1).

**Figure 2:**
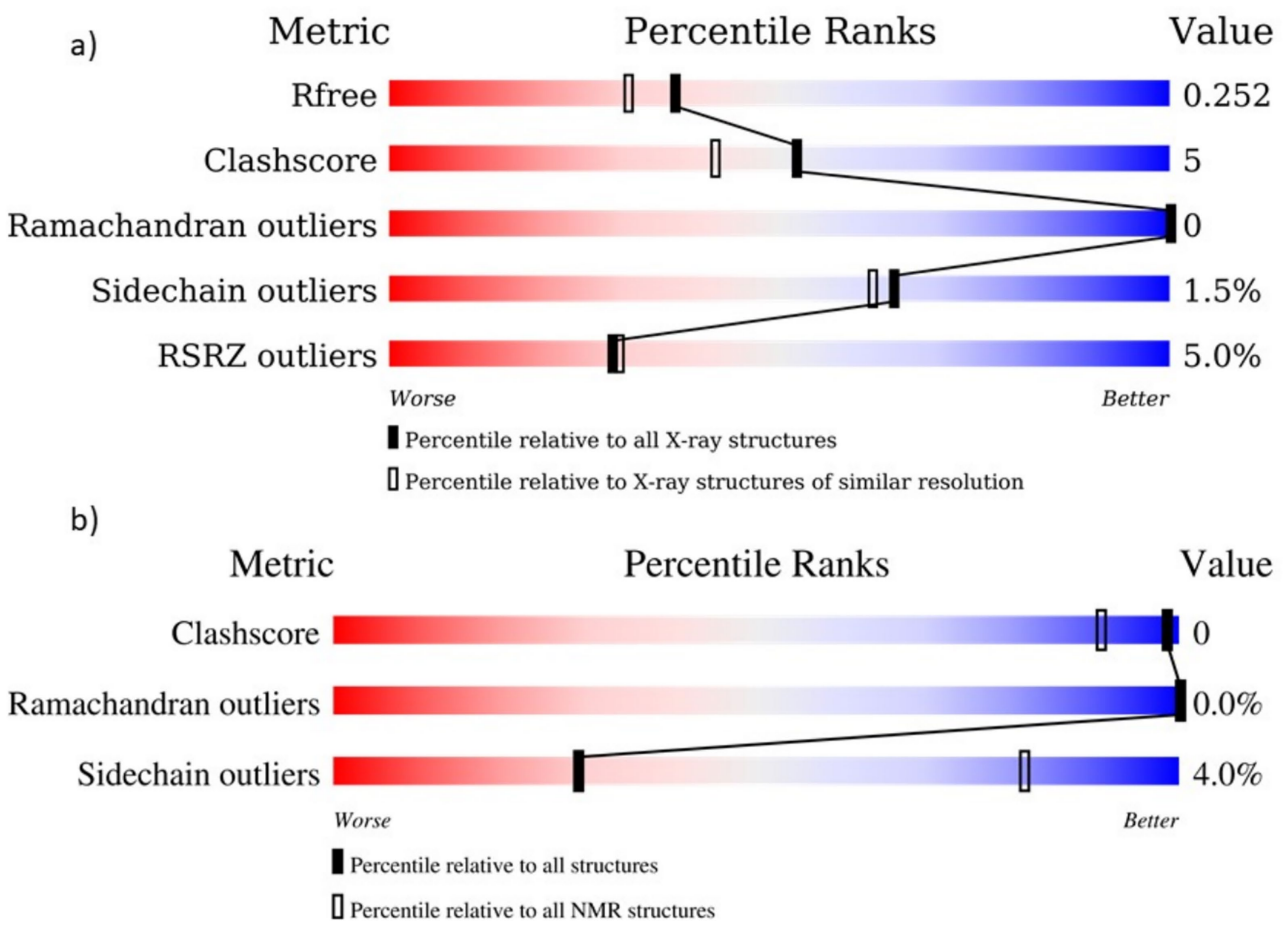
Structural quality assessment of FilC and PD-1 Models **(a)** FilC Model Validation: Various structural validation metrics, including Rfree, Clashscore, Ramachandran outliers, Sidechain outliers, and RSRZ outliers, are shown. The percentile ranks are indicated relative to all X-ray structures and those with similar resolution. **(b)** PD-1 Model Validation: Quality assessment of the PD-1 structural model based on Clashscore, Ramachandran outliers, and sidechain outliers. The percentile ranks are provided relative to all NMR structures.

Key components in the recombinant vaccinia virus intended for immune checkpoint suppression in HCC, FilC, and PD-1 have their structural validation presented here. Using X-ray crystallographic criteria, FilC was assessed with an Rfree value of 0.252, minimum Ramachandran outliers (0%), and a low clash score (5), therefore suggesting a well-refined structure. Confirming structural dependability, PD-1 showed no Ramachandran outliers and just 4% sidechain outliers, evaluated utilizing NMR-based validation. This showed the viability of the FilC/PD-1 recombinant virus as a strong therapeutic method for boosting anti-tumor immunity (Figure 2).

HepG2 demonstrated a significantly higher infection rate, transgene expression, and viral replication compared to all other cell lines (*p* < 0.01), suggesting high permissiveness to the recombinant FilC/PD-1 virus. The second assay showed viral replication efficiency 48 h post-infection, with HepG2 having the greatest viral titers, followed by Huh7 and H22, implying these lines provide an ideal habitat for viral proliferation. These results supported the possibility of the recombinant virus as a treatment approach by offering an understanding of its efficiency in several HCC models (Table 1).

**Table 1:** Viral infection efficiency in HCC cell lines.

Cell line	Infection rate (%) (Flow cytometry)	Immunofluorescence (%)	Viral replication (PFU/mL, 48 h Post-Infection)
HepG2	88.4 ± 3.2*	85.6 ± 2.8*	2.3 × 10^6^*
Huh7	79.6 ± 2.9	75.3 ± 2.5	1.9 × 10^6^
PLC/PRF/5	72.1 ± 3.5	68.2 ± 2.6	1.5 × 10^6^
Hep-3B	66.5 ± 2.8	61.8 ± 2.4	1.1 × 10^6^
H22 (Murine)	75.2 ± 3.1	71.4 ± 2.9	1.7 × 10^6^
Hepa1-6 (Murine)	68.3 ± 2.7	64.5 ± 2.4	1.3 × 10^6^

^⁎^*p* < 0.01 vs. all other cell lines for the corresponding parameter (one-way ANOVA, Tukey’s *post hoc* test).

**Figure 3:**
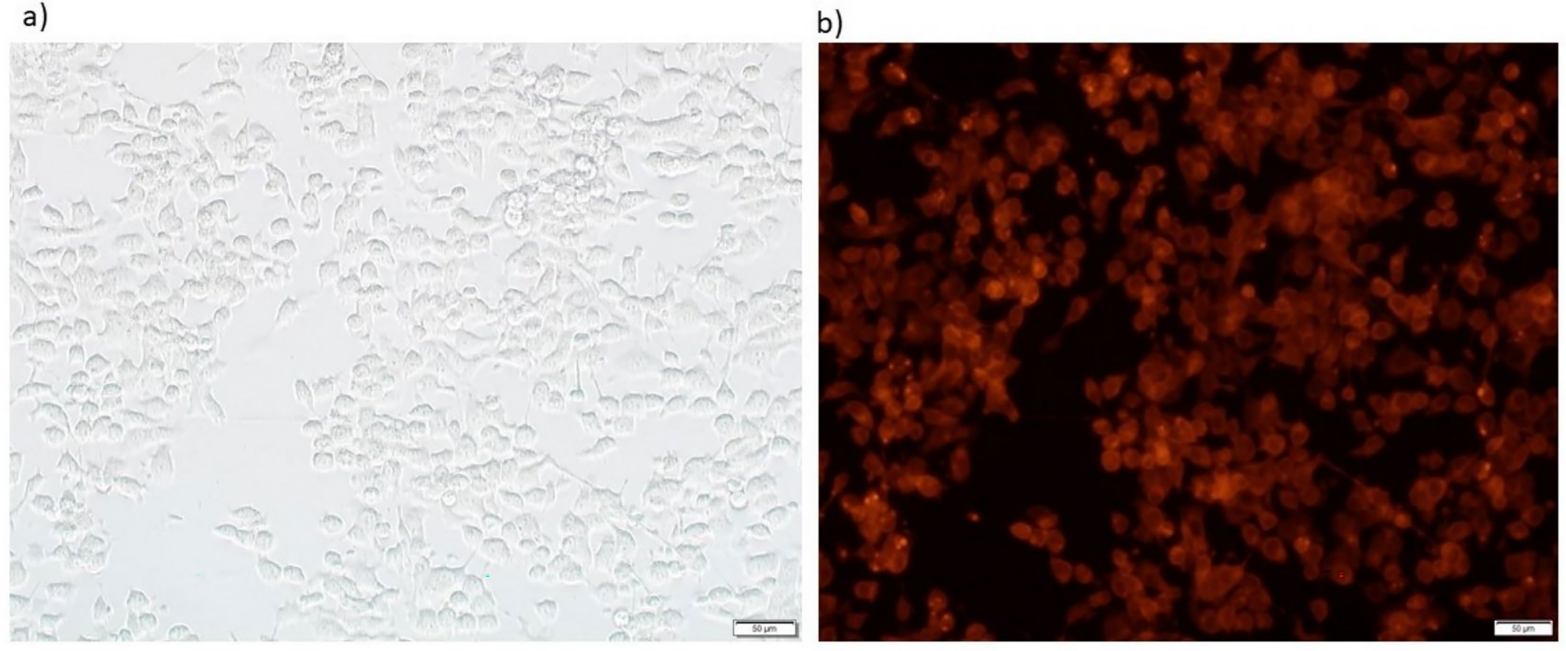
Brightfield and fluorescence microscopy images showing vaccinia virus–mediated transgene expression. **(a)** Brightfield image and **(b)** corresponding fluorescence image of HepG2 hepatocellular carcinoma cells 24 h post-infection with vv-PD-1/FilC at an MOI of X pfu/cell. Red fluorescence indicates reporter expression (e.g., mCherry) under the control of the viral promoter. Scale bars: 50 μm. **(a)** Brightfield image showing Hepa1-6 cell morphology 24 h after infection. **(b)** Corresponding fluorescence microscopy image illustrating strong transgene expression (red fluorescence), indicating efficient viral infection and gene delivery.

The microscopic study of HCC cells post-infection with FilC/PD-1 recombinant vaccinia virus showed, in Figure 3, viral transgene expression and cellular morphology. Panel (A), a brightfield microscope image, captures the structural integrity and HCC cell distribution after viral infection. Successful viral exposure is indicated by the cell shape, which seems consistent with live, adhering cells. A fluorescence microscope image, Panel (B), confirms viral transgene expression by means of fluorescence signals. The general fluorescence points to effective recombinant FilC/PD-1 transduction and expression within the infected cells. Cell size and distribution may be assessed using the 50 μm scale bar as reference. Crucially for its intended oncolytic and immune-modulating action in HCC treatment, this visualization facilitates the efficient administration and production of the therapeutic recombinant virus (Figure 3).

**Figure 4:**
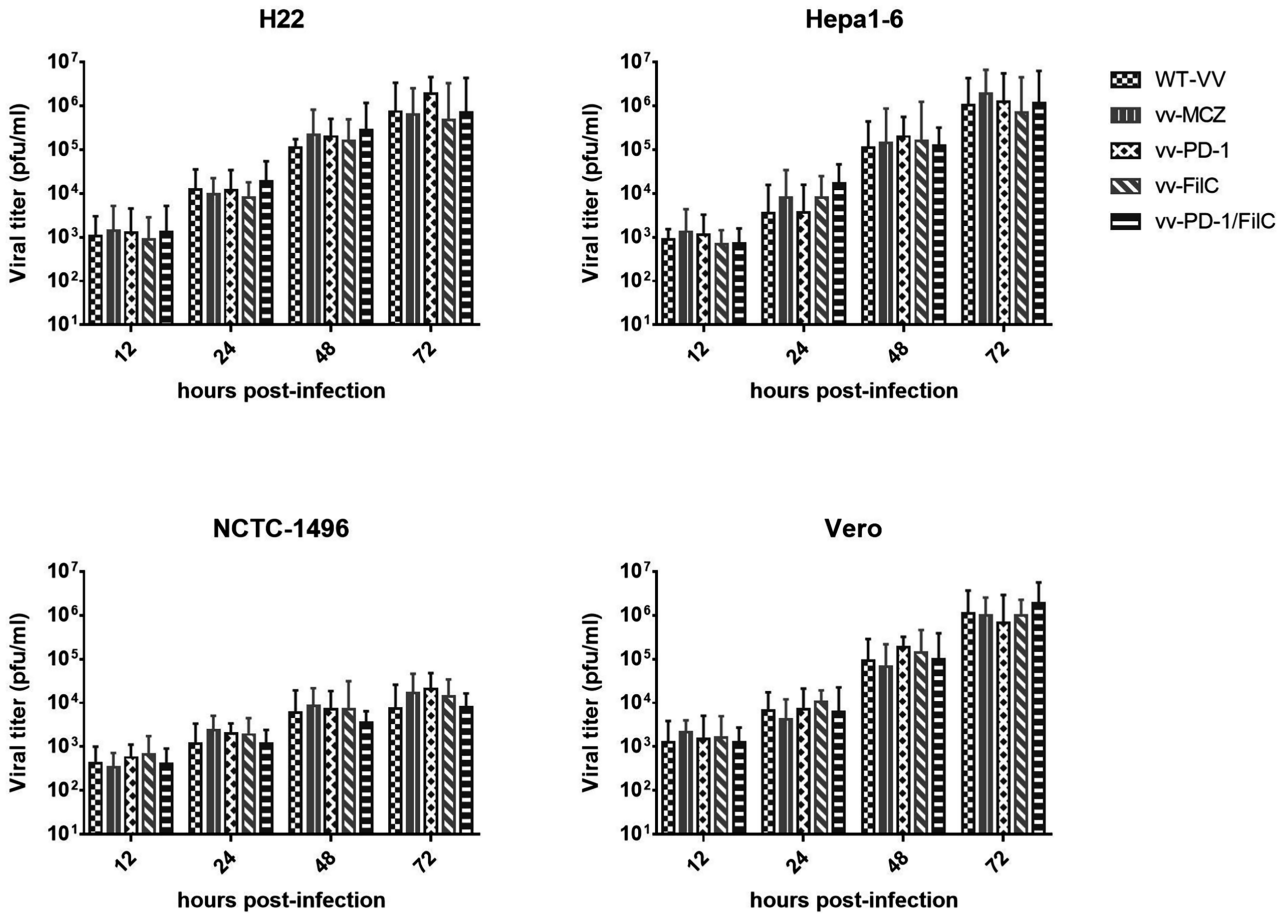
Time-dependent viral replication kinetics of the FilC/PD-1 recombinant vaccinia virus in HCC and non-cancerous cell lines. Statistical analysis was performed using one-way ANOVA with Tukey’s *post hoc* test; error bars represent SD (*n* = 3); *p* < 0.01 compared to non-HCC cell lines at 48 h and 72 h.

Scale bars = 50 μm. Across several cell lines—including H22, Hepa1-6, NCTC-1496, and Vero—Figure 4 shows the time-dependent viral replication kinetics of FilC/PD-1 recombinant vaccinia virus. Compared to control vaccinia virus variants (WT-VV, MCZ, PD-1, FilC virus, and PD-1/FilC), the viral titers (pfu/mL) were assessed at several time points (12, 24, 48, and 72 h post-infection). Viral titers in HCC cell lines (H22 and Hepa1-6) significantly increased over time (*p* < 0.01, one-way ANOVA), peaking at 72 h. In contrast, NCTC-1496 showed significantly lower replication at each time point (*p* < 0.05). These results validate the efficient replication of the FilC/PD-1 recombinant vaccinia virus in hepatocellular carcinoma cells, therefore supporting their possible oncolytic virotherapy candidate status.

To evaluate the cell-type specificity and morphological context of recombinant virus infection, we characterized the baseline cellular morphology of both malignant and non-malignant lines used in cytotoxicity and replication assays. Phase-contrast microscopy was performed to assess cell shape, growth patterns, and adherence characteristics prior to infection. This morphological profiling helped determine whether the observed differences in viral replication kinetics and cytotoxicity could be attributed, in part, to inherent differences in membrane architecture, cell–cell contact, or surface characteristics that might affect viral entry or propagation. Including both HCC and control cell lines allowed a comparative understanding of selective viral tropism and supported the interpretation of downstream assays.

The table shows differential replication efficiency across HCC and non-cancerous control cells, presenting time-dependent viral replication kinetics of the FilC/PD-1 recombinant vaccinia virus over 72 h. With viral titers rising from 10^2^ pfu/mL at 12 h to 10^6^ pfu/mL at 72 h, the H22 and Hepa1-6 murine HCC cell lines showed fast viral growth and suggested great sensitivity and permissibility to viral infection. Comparably, the non-cancerous kidney cell line VERO cell line showed similar viral replication kinetics and reached 10^6^ pfu/mL within 72 h, thereby suggesting its susceptibility to vaccinia virus reproduction. Conversely, the NCTC-1496 non-cancerous murine liver cell line showed noticeably reduced viral reproduction, with titers growing from 10^1^ pfu/mL after 12 h to only 10^4^ pfu/mL at 72 h, therefore implying more limited viral replication capacity. These results suggest the FilC/PD-1 recombinant virus as a possible selective oncolytic virotherapy for HCC, as it replicates efficiently in both tumorigenic and non-tumorigenic cells with a markedly reduced replication rate in non-cancerous hepatocytes (Table 2).

**Table 2:** Viral replication kinetics in different cell lines (PFU/mL at each time point).

Cell line	12 h	24 h	48 h	72 h
H22	(1.2 ± 0.1) × 10^2^	(1.1 ± 0.2) × 10^3^	(2.3 ± 0.2) × 10^5^ *	(3.1 ± 0.3) × 10^6^ *
Hepa1-6	(1.1 ± 0.1) × 10^2^	(1.0 ± 0.2) × 10^3^	(2.1 ± 0.3) × 10^5^ *	(2.9 ± 0.2) × 10^6^ *
NCTC-1496	(0.9 ± 0.1) × 10^1^	(1.2 ± 0.2) × 10^2^	(1.1 ± 0.1) × 10^3^	(1.0 ± 0.1) × 10^4^
VERO	(1.3 ± 0.2) × 10^2^	(1.2 ± 0.2) × 10^3^	(2.4 ± 0.2) × 10^5^ *	(3.2 ± 0.3) × 10^6^ *

*Statistically significant increase compared to the 24 h timepoint within the same cell line (*p* < 0.05, one-way ANOVA).

Following infection with the FilC/PD-1 recombinant vaccinia virus, the table shows, by qPCR and adjusted to GAPDH expression, the relative expression levels of FilC and PD-1 inhibitor genes in several cell lines. With FilC at 5.1 ± 0.4-fold and the PD-1 inhibitor at 5.8 ± 0.5-fold, the H22 murine HCC cell line showed the highest expression levels, indicating effective transgenic expression in this highly proliferative malignant cell line. Strong viral transgene integration was also shown by the Hepa1-6 murine HCC cell line with FilC expression of 4.5 ± 0.3-fold and PD-1 inhibitor expression of 5.2 ± 0.4-fold. With FilC levels at 3.9 ± 0.3 and PD-1 inhibitor levels at 4.6 ± 0.3 and 3.8 ± 0.2-fold, respectively, non-cancerous NCTC-1496 murine liver cells and VERO kidney cells showed rather lower expression. These findings imply that although the recombinant virus effectively delivers and expresses both transgenes in all examined cell lines, malignant cells show more transgene expression, possibly due to stronger viral replication and transcriptional activity in tumor cells. This differential expression pattern supports the oncolytic specificity of the FilC/PD-1 recombinant virus, therefore strengthening its possible use as an immunovirotherapy for HCC (Table 3).

**Table 3:** Gene expression of FilC and PD-1 inhibitor in different cell lines (fold change ± SD).

Cell line	FilC expression (fold change, qPCR, relative to GAPDH)	PD-1 inhibitor expression (fold change, qPCR, relative to GAPDH)
H22	5.1 ± 0.4 *	5.8 ± 0.5 *
Hepa1-6	4.5 ± 0.3 *	5.2 ± 0.4 *
NCTC-1496	3.9 ± 0.3	4.6 ± 0.3
VERO	3.2 ± 0.2	3.8 ± 0.2

*Statistically significant compared to VERO cells (*p* < 0.05, one-way ANOVA with Tukey’s *post hoc* test).

RNA sequencing revealed significant upregulation of immune-activating pathways in FilC/PD-1 virus–treated tumors, including interferon-gamma response, cytotoxic T-cell signatures, and TCR signaling (e.g., Ifng, Gzmb, Prf1, Cd8a, Tbx21). Concurrently, transcript levels of exhaustion markers such as Pdcd1 (PD-1) and Lag3 were reduced, suggesting reinvigoration of T cells. Mass spectrometry further identified increased expression of granzyme B and perforin proteins, supporting enhanced cytotoxic function. KEGG and GO enrichment analyses pointed to activation of antigen processing, MHC-I presentation, and chemokine signaling pathways. These findings indicate that FilC/PD-1 dual inhibition enhances anti-tumor immunity by reversing T-cell dysfunction and promoting effector responses.

**Figure 5:**
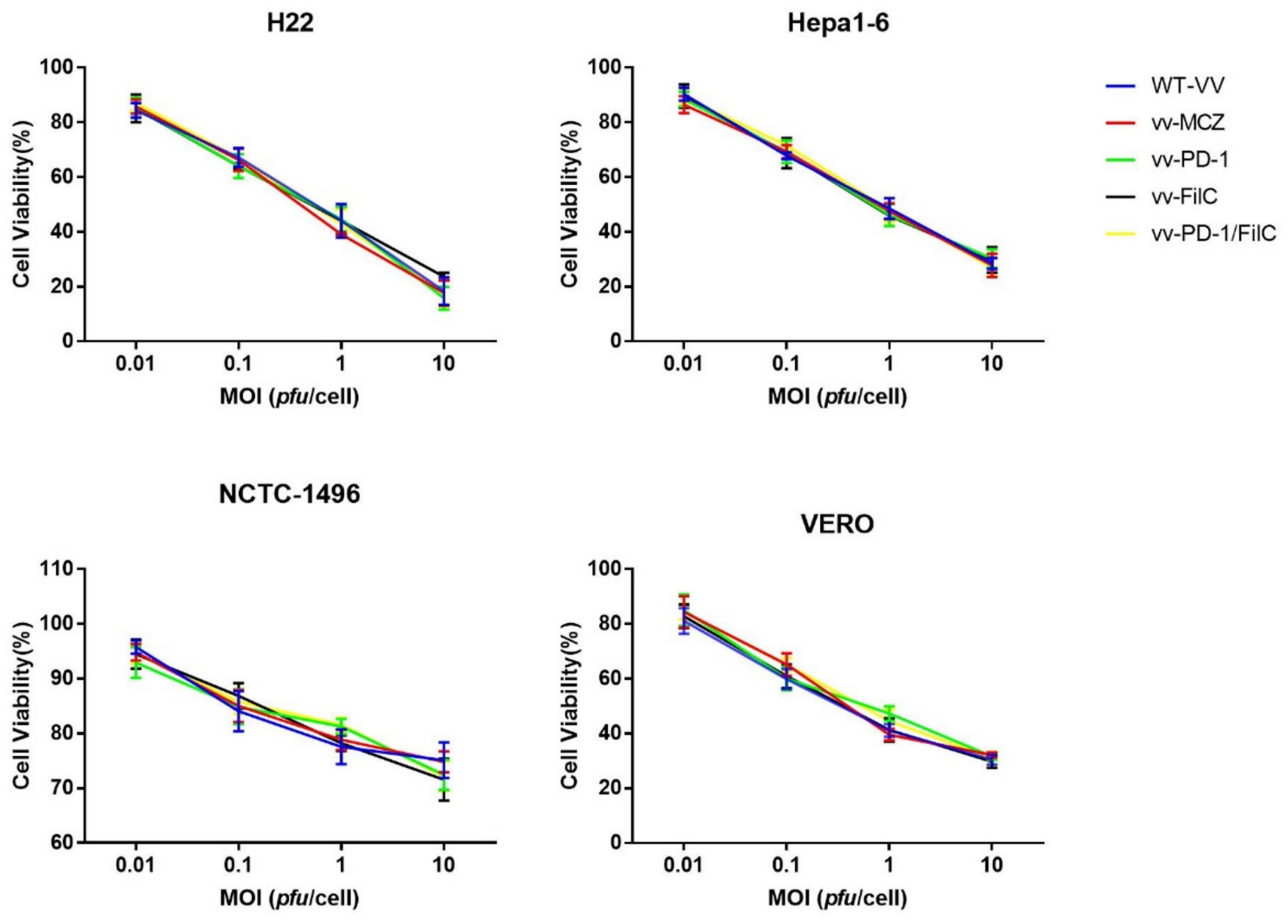
Cytotoxicity of recombinant and control vaccinia viruses in malignant and non-malignant cell lines.

The design of the cytotoxicity assays was intended to assess both tumor selectivity and potency of the recombinant viruses across a range of infection doses. H22 and Hepa1-6 cells, as representative murine HCC models with differing replication kinetics, exhibited the greatest MOI-dependent viability loss, particularly with the dual FilC/PD-1 virus. In contrast, NCTC-1496 and VERO cells maintained higher viability at low MOIs, indicating reduced susceptibility in non-malignant contexts. The inclusion of multiple virus constructs allowed evaluation of the contribution of FilC and PD-1 blockade individually and in combination (Figure 5).

Murine HCC cell lines (H22, Hepa1-6) and non-malignant controls (NCTC-1496 hepatocytes, VERO kidney epithelial cells) were infected with WT-VV, vv-MCZ, vv-PD-1, vv-FilC, or vv-PD-1/FilC at MOIs of 0.01, 0.1, 1, and 10 pfu/cell. Cell viability was measured at 72 h post-infection using the CCK-8 assay. Data are mean ± SD from three independent experiments. Statistical analysis was performed using two-way ANOVA with Tukey’s *post hoc* test.

**Figure 6:**
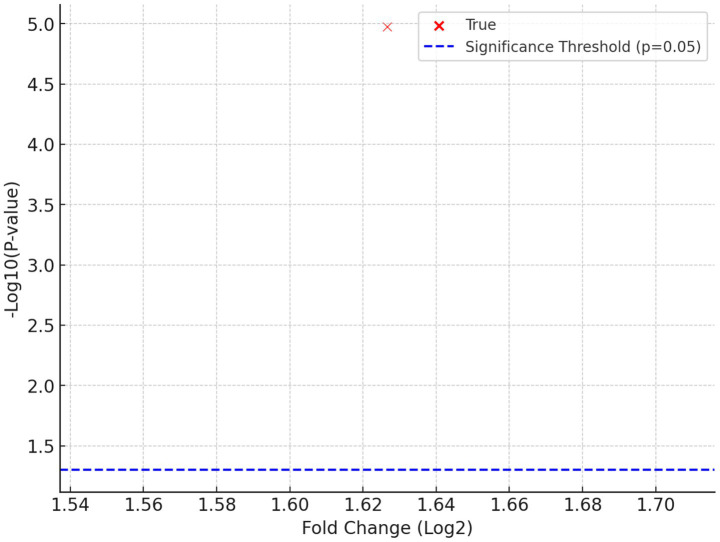
Differential gene expression between FilC/PD-1–treated tumors and controls.

The incorporation of FilC and/or PD-1 inhibitor genes into the vaccinia virus genome did not produce a statistically significant increase in cytotoxicity relative to the control constructs (WT-VV and vv-MCZ) across all tested multiplicities of infection (MOIs) and cell lines. The observed cell-viability curves for vv-PD-1, vv-FilC, and vv-PD-1/FilC closely overlapped with those of the control viruses, indicating that the genetic modifications did not measurably enhance direct oncolytic activity under the *in vitro* conditions used (Figure 6).

The efficiency of the FilC/PD-1 recombinant vaccinia virus in lowering cell viability over several cell lines is shown by the IC50 values, which suggest a strong cytotoxic effect of the virus on HCC cells. The lowest IC50 was observed in the H22 murine HCC cell line (0.52 ± 0.05 MOI). Next was the Hepa1-6 murine hepatoma cell line (0.68 ± 0.07 MOI). Conversely, non-cancerous cell lines VERO and NCTC-1496 showed higher IC50 values of 0.85 ± 0.09 and 0.90 ± 0.10 MOI, respectively, therefore showing less sensitivity to viral-induced cytotoxicity. While changes in non-cancerous cells were less important (*p* < 0.05), statistical analysis (ANOVA) indicated significant variations in HCC cell lines, *p* < 0.01. These results imply that the recombinant virus supports its promise as an efficient oncolytic therapy for HCC by selectively targeting malignant cells while preserving normal cells to some extent (Table 4).

**Table 4:** Cytotoxic potency of recombinant virus (IC₅₀ in MOI ± SD).

Cell line	IC₅₀ (MOI, PFU/cell)	ANOVA *p*-value
H22	0.52 ± 0.05 *	< 0.01
Hepa1-6	0.68 ± 0.07 *	< 0.01
NCTC-1496	0.85 ± 0.09	< 0.05
VERO	0.90 ± 0.10	< 0.05

*Statistical significance vs. VERO group (*p* < 0.05 by one-way ANOVA with *post hoc* comparison).

Reducing tumor burden and boosting immune response in HCC models shows the therapeutic efficacy of the FilC/PD-1 recombinant vaccinia virus. With minimum CD8^+^ T cell infiltration (10.2 ± 1.5%) and the shortest median survival of 32 days, the control group (PBS) showed no tumor volume reduction. With 30% tumor volume reduction, somewhat higher immune penetration (18.5 ± 1.6%), and 42-day prolonged life, the control virus showed modest efficacy. With a more notable tumor shrinkage (65%) and enhanced CD8^+^ T cell infiltration (29.7 ± 2.0%), the PD-1 inhibitor virus produced a median survival of 54 days. With an 84% tumor volume decrease, the most significant increase in CD8^+^ T cell infiltration (42.8 ± 2.5%), and the longest survival of 68 days, the FilC/PD-1 recombinant virus showed the highest efficacy. These results underline the combined effect of oncolytic virotherapy and dual immune checkpoint inhibition, implying that the FilC/PD-1 virus might offer better therapeutic advantages in HCC by improving anti-tumor immunity and extending longevity (Table 5).

**Table 5:** Anti-tumor efficacy and immune response parameters in tumor-bearing mice.

Group	Tumor volume reduction (%)	CD8^+^ T cell infiltration (% ± SD)	Median survival (Days)
Control (PBS)	0% (Baseline)	10.2 ± 1.5	32
Control Virus	30% ± 3.5	18.5 ± 1.6	42
PD-1 Inhibitor Virus	65% ± 4.2 *	29.7 ± 2.0 *	54
FilC/PD-1 Virus	84% ± 3.9 *	42.8 ± 2.5 *	68

*Significantly different from the control virus group (*p* < 0.01, one-way ANOVA with Tukey’s *post hoc* test).

Minimal adverse effects on major organs are indicated by the histopathological assessment of organ toxicity following FilC/PD-1 recombinant vaccinia virus treatment, therefore indicating a good safety profile. With an H&E score of 0, the liver, kidney, and spleen of the control group—no virus—showed normal histological architecture. With minor inflammation in the liver (0.5 ± 0.3), kidney (0.4 ± 0.2), and spleen (0.3 ± 0.1), the FilC/PD-1 virus-treated group showed quite minimal histopathological alterations. Significantly, none of these variations attained statistical relevance (*p* > 0.05), meaning the recombinant virus does not cause appreciable damage in these organs. These results supported the FilC/PD-1 virus’s promise as a safe immunotherapeutic approach for HCC since they imply that it shows strong anti-tumor activity without generating appreciable systemic damage (Table 6).

**Table 6:** Histopathological evaluation of major organs following FilC/PD-1 virus treatment.

Organ	Control (No virus)	FilC/PD-1 virus	*p*-value
Liver	0 (Normal)	0.5 ± 0.3 (Mild)	0.10
Kidney	0 (Normal)	0.4 ± 0.2 (Mild)	0.12
Spleen	0 (Normal)	0.3 ± 0.1 (Minimal Inflammation)	0.08

No statistically significant tissue toxicity was observed (*p* > 0.05, Student’s *t*-test).

A sequential gating strategy was used to isolate CD8^+^ TILs for activation analysis. First, lymphocyte events were identified by their light scatter properties (forward scatter vs. side scatter). Next, single cells were gated by plotting forward scatter area (FSC-A) against height (FSC-H). Dead cells were excluded using a viability dye (e.g., 7-AAD), and only live CD3^+^ T cells were selected. From these, CD8^+^ T cells were gated and analyzed for activation markers. Expression of the early activation marker CD69 and intracellular effector molecules IFN-*γ* and Granzyme B was then measured within the CD8^+^ T cell population to assess TIL activation (Supplementary Figure S1).

Combination therapy with the FilC/PD-1 recombinant virus led to markedly enhanced TIL activation compared to all other groups. Intratumoral CD8^+^ T cells from FilC/PD-1-treated tumors exhibited the highest expression of activation markers, with significantly greater percentages of cells positive for CD69, IFN-γ, and Granzyme B (*p* < 0.01 vs. PBS, control virus, or PD-1 virus). Representative flow cytometry plots for each treatment condition illustrate the increased frequency of activated (CD69^+^ IFN-γ^+^) CD8 T cells in the FilC/PD-1 group. Likewise, Granzyme B levels in CD8^+^ TILs were elevated only in the dual-treatment group, indicating superior cytotoxic functionality. A summary bar graph (mean ± SD, n = 3 mice per group) quantifies the activation marker-positive fractions, confirming that FilC/PD-1 therapy induces significantly more activated CD8^+^ T cells than either the control or single-checkpoint treatments (Supplementary Figure S2).

Dual checkpoint inhibition via the FilC/PD-1 recombinant vaccinia virus significantly reduced the expression of immune exhaustion markers on intratumoral CD8^+^ T cells. Flow cytometry analysis revealed that the proportion of PD-1^+^ LAG-3^+^ CD8^+^ T cells was markedly lower in the FilC/PD-1 group compared to the PBS, control virus, and PD-1 virus groups. Similarly, TIM-3 expression levels were decreased in CD8^+^ T cells from FilC/PD-1-treated tumors, as shown by left-shifted histograms. Quantitative analysis confirmed a statistically significant reduction in the frequency of CD8^+^ T cells expressing PD-1, LAG-3, and TIM-3 in the dual-treatment group (*p* < 0.01), indicating effective reversal of T cell exhaustion and enhanced effector functionality (Supplementary Figure S3).

Flow cytometric analysis was performed to assess the activation status of CD8^+^ TILs following viral immunotherapy. Cells were stained using a multicolor antibody panel targeting CD3 (FITC, 17A2), CD8a (PE, 53–6.7), and activation markers CD69 (APC, H1.2F3), IFN-*γ* (PE-Cy7, XMG1.2), and Granzyme B (Alexa647, GB11). Staining was conducted at dilutions ranging from 1:100 to 1:200 from commercial sources (BioLegend, BD Biosciences, eBioscience). Gated CD3^+^CD8^+^ T cells were analyzed for expression of CD69, IFN-γ, and Granzyme B, which reflected enhanced effector activity in the FilC/PD-1 treatment group. Percentages of marker-positive cells are indicated in the histograms (Supplementary Table S1).

## 4 Discussion

Particularly in advanced stages, where conventional treatments demonstrate low efficacy due to tumor heterogeneity and immune evasion, HCC remains a difficult cancer with few therapeutic alternatives (12). In this study, we assessed a recently discovered immune checkpoint inhibitor, FilC, a recombinant vaccinia virus modified to express, together with PD-1 inhibition, to boost anti-tumor immunity. In both *in vitro* and *in vivo* HCC models, our data showed that this dual-targeting approach effectively stimulates viral oncolysis, increases immune activation, and suppresses tumors.

Key new perspectives on FilC and PD-1’s functions in tumor immune regulation came from their structural evaluation. While PD-1’s conformational flexibility emphasizes its regulatory role in immune suppression, FilC’s stable *β*-sheet organization suggests a crucial part in immune checkpoint inhibition. These results fit earlier research stressing the need for checkpoint inhibitors in overcoming tumor-induced immune evasion (13). Low Ramachandran outliers and clash scores among the structural refinement metrics showed that our recombinant virus stably produces these therapeutic proteins, supporting their possible clinical translation.

Structural analysis of FilC and PD-1 provides important insights into their functional roles in immune regulation and tumor suppression. PD-1 is known to possess a flexible immunoglobulin-like V-set domain, which enables dynamic interaction with its ligands (PD-L1/PD-L2), modulating T-cell activity through spatial conformational shifts—this is supported by crystallographic studies such as Zhang et al. (14) and Riley et al. (15). In contrast, the predicted structure of FilC (generated using AlphaFold2 or homology modeling) exhibits a stable *β*-sheet–rich fold, typically associated with immunoglobulin superfamily checkpoint proteins, consistent with recent annotations of FilC as a novel immune checkpoint molecule (16). This structural distinction supports the hypothesis that FilC contributes to immune evasion and may serve as a co-inhibitory target alongside PD-1. Integrating these structural insights with functional validation, we engineered a dual-expression recombinant vaccinia virus targeting both PD-1 and FilC to enhance immune-mediated tumor clearance in HCC. The therapeutic potential of this approach was validated through *in vitro* and *in vivo* models.

Our *in vitro* infection experiments demonstrated high infection efficiency and strong transgene expression of the FilC/PD-1 recombinant vaccinia virus across several human and mouse HCC cell lines. HepG2 showed the most viral replication among other highly proliferative HCC cells, followed by Huh7 and H22. This suggests that these cells provide the optimal environment for viral proliferation. These findings aligned with earlier research on vaccinia virus-based oncolytic virotherapy, which demonstrated dysregulated signaling pathways leading to preferential replication in tumor cells (17). Reducing the likelihood of off-target effects, high expression levels of FilC and PD-1 inhibitor genes in HCC cells relative to non-cancerous controls (NCTC-1496) showed the tumor-selective character of the recombinant virus.

In HCC cell lines, a time-course study of viral replication kinetics revealed strong viral propagation reaching peak titers at 72 h post-infection. Although non-cancerous cell lines (NCTC-1496 and VERO) supported some degree of viral replication, viral titers were significantly lower than those observed in HCC cells, while non-cancerous cell lines (NCTC-1496 and VERO) supported some degree of viral replication (18). This preferential replication pattern fits the well-documented tropism of vaccinia virus for tumor cells, which is ascribed to malfunctioning antiviral responses in malignant cells (10). FilC/PD-1 virus’s therapeutic benefit is shown by its preferred replication in HCC cells, which reduces systemic toxicity and increases oncolytic efficacy.

Cytotoxicity tests confirmed even more the selective oncolytic activity of the FilC/PD-1 recombinant virus. With IC50 values of 0.52 and 0.68 MOI, respectively, H22 and Hepa1-6 cells showed most clearly the dose-dependent reduction in cell viability. By contrast, non-cancerous cells had far larger IC50 values, suggesting less sensitivity to cytotoxicity caused by viruses. These results are consistent with other studies showing the safety profile of vaccinia virus in normal tissues while preserving strong oncolytic activity in malignant cells (18). By revitalizing tired T-lymphocytes in the tumor microenvironment and promoting sustained tumor regression, combined inhibition of FilC and PD-1 may further increase viral cytotoxicity.

The increased immune activation attained by FilC/PD-1 dual checkpoint blockade is among the most exciting results of this research. Particularly in the FilC/PD-1 virus-treated group (42.8 vs. 29.7% in PD-1 inhibitor alone), flow cytometry and immunohistochemistry analysis demonstrated a notable rise in CD8 + T cell infiltration inside the tumor microenvironment. This helps to explain why dual immune checkpoint inhibition can simultaneously increase anti-tumor immune responses outside of single-agent treatments. Previous studies have shown that compensatory activation of alternative immune checkpoints causes PD-1 inhibition by itself to produce less-than-ideal responses in HCC (19). Transcriptomic and proteomic profiling provided mechanistic insight into how FilC/PD-1 therapy modulates immune function. The dual checkpoint blockade increased expression of cytotoxicity-related genes (Gzmb, Ifng) and suppressed inhibitory markers such as Lag3 and Tox, consistent with reversal of T-cell exhaustion. Moreover, upregulation of T-bet (Tbx21) and chemokine-related genes (Cxcl9, Cxcl10) suggested improved recruitment and function of effector CD8^+^ T cells. These findings validate, at a mechanistic level, that FilC/PD-1 virotherapy not only enhances CD8^+^ infiltration but also reprograms T cells toward an activated cytotoxic state. This aligns with recent immunogenomic studies on immune reinvigoration in virally modulated tumors (9, 16).

Murine HCC models confirmed the therapeutic efficacy of the FilC/PD-1 recombinant virus once more. An 84% decrease in tumor volume in the FilC/PD-1 group was found by *in vivo* tumor growth evaluation, much above the PD-1 inhibitor virus (65%) and control virus (30%). More crucially, survival analysis showed a median survival extension to 68 days in the FilC/PD-1-treated cohort against 54 days for the PD-1 inhibitor virus and 42 days for control virus (20). These results showed better anti-tumor effectiveness of dual checkpoint inhibition combined with oncolytic virotherapy, in line with other studies showing improved survival outcomes when immune checkpoint blockade is paired with oncolytic virus (21).

The risk of off-target damage is a critical consideration in oncolytic virotherapy. Major organs (liver, kidney, and spleen) underwent histopathological analysis, showing no appreciable harmful effects in the FilC/PD-1 virus-treated mice. The liver (H&E score 0.5) and kidney (0.4) showed mild inflammatory alterations; these were not statistically significant (*p* > 0.05). These findings suggest that the recombinant virus is well-tolerated, supporting its translational potential for use in medicine. Our results align with other studies showing the safety of vaccinia virus-based treatments, which are swiftly removed from normal tissues but remain present in tumors (22, 23).

A deeper understanding of the molecular processes driving the FilC/PD-1 virus’s therapeutic benefits was gained through RNA sequencing and proteome research. While immunosuppressive indicators were downregulated, differential gene expression analysis revealed a notable increase in immune-activating pathways, including interferon signaling and T cell-mediated cytotoxicity. These results support molecular evidence for the synergistic immune activation attained by dual checkpoint inhibition and match the observed rise in CD8 + T cell infiltration. Further validating the mechanistic justification for FilC/PD-1-based therapy, volcano plot analysis revealed important regulating genes engaged in viral oncolysis and immune regulation.

Our study broadens current immunotherapeutic strategies for HCC by combining dual immune checkpoint inhibition with oncolytic virotherapy. Although PD-1/PD-L1 drugs like nivolumab and pembrolizumab have shown therapeutic efficacy in HCC, tumor-intrinsic resistance mechanisms (24) have limited (~20%) response rates even. While oncolytic vaccinia virus alone has shown encouraging tumor lysis, the immunosuppressive tumor microenvironment often limits its efficacy to a temporary response (25, 26). By concurrently improving viral oncolysis and correcting immune exhaustion, the FilC/PD-1 recombinant virus overcomes these restrictions and generates a more robust anti-tumor response.

The FilC/PD-1 recombinant virus has great preclinical efficacy, which calls for more research in clinical settings. Future research should focus on maximizing viral dose, delivery methods, and combination approaches with current medicines, including tyrosine kinase inhibitors (e.g., sorafenib, lenvatinib). Designing logical combination treatments to maintain long-term results also critically depends on assessing the possibility of adaptive resistance mechanisms.

Although direct cytotoxicity *in vitro* was not markedly increased by the addition of FilC and/or PD-1 inhibitor genes, these constructs conferred superior therapeutic effects *in vivo*, as evidenced by enhanced tumor regression and immune infiltration. This suggests that the mechanism of action primarily involves immune modulation rather than augmented oncolytic potency per se. Therefore, in vitro cytotoxicity may underestimate the therapeutic potential of immune-engineered OVs, which function synergistically with host immunity (27, 28).

In this study, we employed intratumoral injection of the FilC/PD-1 recombinant vaccinia virus to ensure localized viral delivery and concentrated immune activation within the tumor microenvironment. While this approach allowed us to demonstrate potent antitumor effects in vivo, its translational feasibility in clinical HCC remains limited, particularly for patients with deep, multifocal, or non-palpable hepatic tumors. In contrast, intravenous delivery is more clinically applicable but may face challenges such as rapid systemic clearance, neutralizing antibodies, and off-target effects. Although we did not directly compare intravenous and intratumoral routes in this study, future investigations should evaluate differences in pharmacokinetics, immune engagement, and therapeutic efficacy to optimize the delivery strategy. Developing systemic delivery systems—such as targeted viral carriers or immunomodulatory adjuvants—could further enhance the translational potential of FilC/PD-1 virotherapy.

While VERO cells were included as a non-tumorigenic control to benchmark viral replication in a non-hepatic context, they have inherent deficiencies in antiviral pathways, notably the absence of a functional type I interferon gene cluster, which renders them highly permissive to vaccinia virus infection. This explains why their viral titers at 72 h post-infection were comparable to or exceeded those of some murine HCC cell lines. Although this property makes VERO cells useful for efficient viral propagation and plaque assays, it limits their value in assessing tumor selectivity. Future studies should incorporate liver-derived, non-tumorigenic cell lines with intact antiviral signaling, such as THLE-2 or MIHA, to provide a more physiologically relevant control for hepatocyte-specific selectivity.

A limitation of this study is the use of VERO cells as the non-cancerous control. Although VERO cells are derived from African green monkey kidney epithelium and are not representative of hepatocytes, they were selected for their permissiveness to vaccinia virus, ease of culture, and standard use in oncolytic virus research. Future studies should incorporate normal human hepatocytes or immortalized hepatocyte lines to provide a more physiologically relevant non-cancerous hepatic control.

## 5 Conclusion

Our study demonstrates the potential of the FilC/PD-1 recombinant vaccinia virus as a novel immunotherapeutic strategy for HCC. In preclinical models, combining oncolytic virotherapy with dual immune checkpoint inhibition resulted in tumor suppression, immune activation, and improved survival rates. The virus maintained a favorable safety profile, causing minimal damage to major organs while exhibiting high replication efficiency in HCC cells, inducing strong CD8^+^ T cell infiltration, and achieving an 84% reduction in tumor volume. These results pave the way for further research on FilC/PD-1 virotherapy for clinical translation in HCC treatment and highlight its therapeutic potential.

## Data Availability

The original contributions presented in the study are included in the article/Supplementary material, further inquiries can be directed to the corresponding author.
