# Supplementary figures and images for: Novel immune checkpoint inhibitor FilC/PD-1 recombinant vaccinia virus inhibits hepatocellular carcinoma
Source: Front Med (Lausanne). 2025 Sep 8;12:1622209. doi: 10.3389/fmed.2025.1622209 (PMC12450999; doi:10.3389/fmed.2025.1622209)

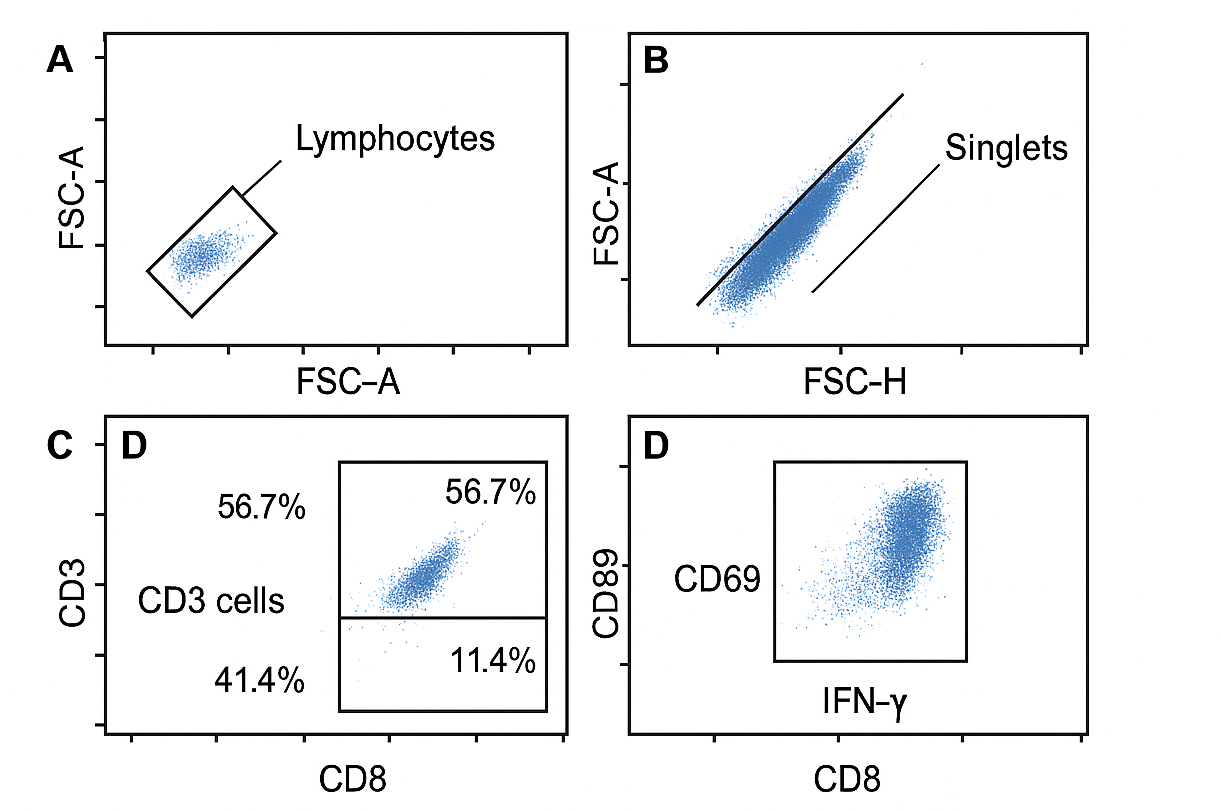

Supplement: Supplementary file 1 [file Image_1.PNG]

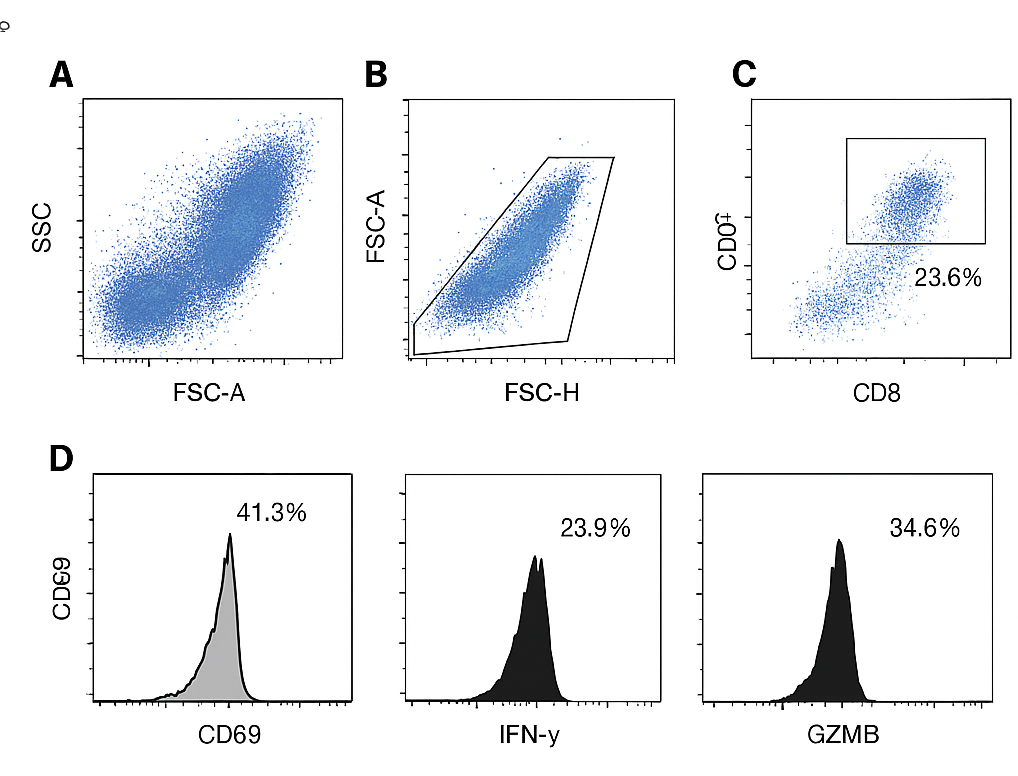

Supplement: Supplementary file 2 [file Image_2.PNG]

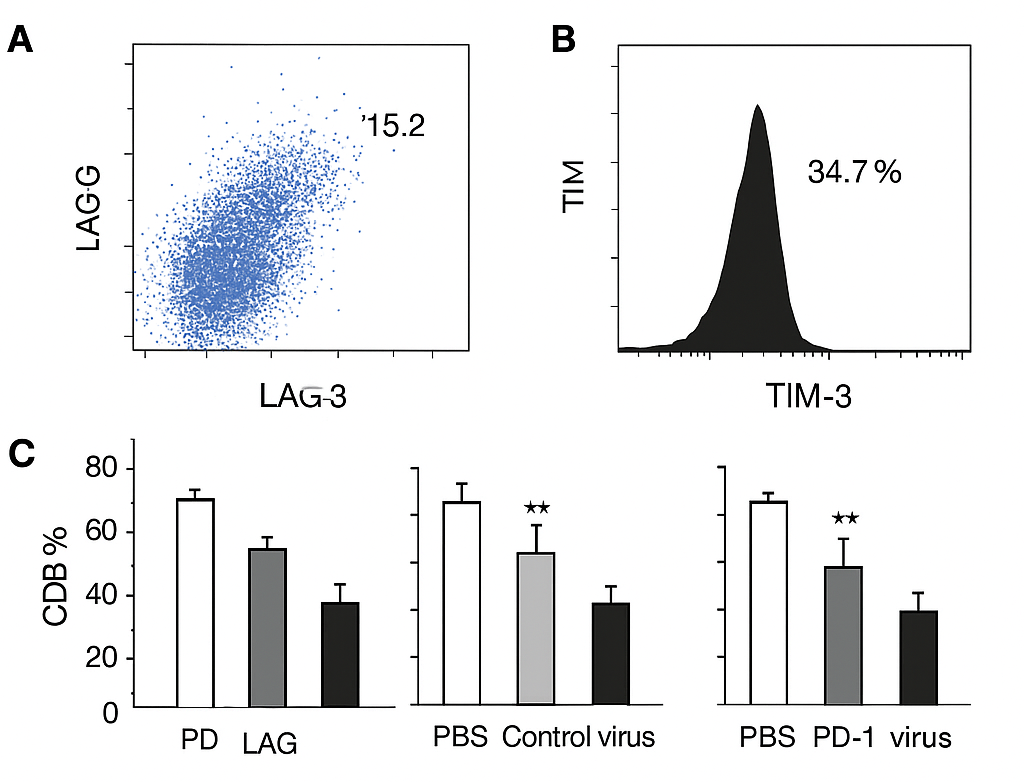

Supplement: Supplementary file 3 [file Image_3.PNG]
